# Combined treatment of malignant salivary gland tumours with intensity-modulated radiation therapy (IMRT) and carbon ions: COSMIC

**DOI:** 10.1186/1471-2407-10-546

**Published:** 2010-10-11

**Authors:** Alexandra D Jensen, Anna Nikoghosyan, Christine Windemuth-Kieselbach, Jürgen Debus, Marc W Münter

**Affiliations:** 1 Dept. of Radiation Oncology, INF 400, 69120 Heidelberg, Germany; 2 Alcedis GmbH, Winchester-Str. 2, 35394 Gießen, Germany

## Abstract

**Background:**

Local control in malignant salivary gland tumours is dose dependent. High local control rates in adenoid cystic carcinomas could be achieved by highly conformal radiotherapy techniques and particle (neutron/carbon ion) therapy. Considering high doses are needed to achieve local control, all malignant salivary gland tumours probably profit from the use of particle therapy, which in case of carbon ion treatment, has been shown to be accompanied by only mild side-effects.

**Methods/design:**

The COSMIC trial is a prospective, mono-centric, phase II trial evaluating toxicity (primary endpoint: mucositis ≥ CTCAE°3) and efficacy (secondary endpoint: local control, disease-free survival) in the combined treatment with IMRT and carbon ion boost in 54 patients with histologically proved (≥R1-resected, inoperable or Pn+) salivary gland malignancies. Patients receive 24 GyE carbon ions (8 fractions) and IMRT (50 Gy at 2.0 Gy/fraction).

**Discussion:**

The primary objective of COSMIC is to evaluate toxicity and feasibility of the proposed treatment in all salivary gland malignancies.

**Trial Registration:**

Clinical trial identifier NCT 01154270

## Background

Malignant salivary gland tumours are rare and a heterogenous group of tumours accounting for about 3-5% of head and neck cancers. High-grade tumours such as mucoepidermiod carcinoma (35%) and adenoid cystic carcinoma (25%) are the most common histological subtypes [1], characterised by a rather slow pattern of growth, perineural spread and high propensity for haematogenous metastases. So far, standard therapy of high-grade salivary gland carcinoma consists of complete surgical resection and adjuvant radiation in high-risk situations (R+ or close margin, perineural spread, neural infiltration, large tumours (T3/4), or nodal metastases [2-4]. To achieve local control, radiation doses of > 60 Gy or even 66 Gy (5) are recommended [5,6], with apparently all tumour stages profiting from postoperative radiotherapy [5,7-9].

Regarding radiation therapy, local control was also significantly improved by the application of high-precision techniques, dose-escalation and high-LET radiation [9-13].

Intensity-modulated radiation therapy (IMRT) as well as fractionated stereotactic RT could already improve local control as compared to conventional RT techniques achieving 3-year PFS rates of 38% [14].

So far, the highest local control rates at 75 - 100% [11,13] were achieved by neutron radiation albeit at the cost of significant late toxicity.

Dose escalation [12] though only in a small group of patients, in treatment with heavy ions at 70.2 GyE (3 × 3.9 GyE/wk) or 64 GyE (4 × 4 GyE/wk) was shown to be feasible and beneficial. Despite high doses per fraction, no CTC °III late toxicities and very few °III acute reactions occurred. Local control at 5 years was still 100%, however, various histological subtypes of salivary gland tumours were included in the trial. Pommier et al. [15] treated 23 patients with adenoid cystic carcinoma with protons at 75,9 GyE (median) in various fractionation schemes. Overall local control at 5 years was 93%, however, the authors noted several °III as well as one °V late toxicity (temporal lobe necrosis). Douglas et al [11] published a retrospective analysis of 159 patients with adenoid cystic carcinoma treated with neutrons (total dose: 19,2 Gy), but only reported somewhat disappointing local control rates of 57% at 5 years accompanied with significant late toxicity (14% > CTC °III). Results of the combined IMRT-C12 treatment therefore compare favourably [10] with only very mild treatment-related side effects (°III late tox in only 1 patient) [16,17]. 54 Gy IMRT (2 Gy/Fx) plus 18 GyE C12-boost (3 GyE/Fx) yielded local control rates of about 78% at 4 years [10].

In view of the outcome and low toxicity profile, photon IMRT plus C12-boost has been accepted as the standard treatment and method of choice for adenoid cystic carcinoma in Germany. Due to the possibility to apply higher doses at so far mild side effects, charged-particle therapy does promise improved results for all types of malignant salivary gland cancers.

Therefore, this trial basically evaluates relative dose escalation with simultaneous normal tissue sparing by using a combination of C12-heavy ion therapy and IMRT in all salivary gland malignancies including mucoepidermoid carcinoma and adenoid cystic carcinoma. Looking at other head and neck tumors i.e. squamous cell carcinoma of the head and neck (SCCHN), radiation therapy commonly causes pronounced mucositis. With therapy usually about 7 weeks duration and mucositis, improvement of clinical symptoms can only be expected at least 2-3 weeks post completion of therapy. At the occurrence of mucositis CTC °III, pain, loss of taste, and dysphagia causing reduced food/fluid intake and weight loss can potentially cause treatment delays, protraction of treatment and worse outcome. Therefore, evaluation of this particular side effect was chosen as the primary endpoint of the trial. Trial objectives hence assess accompanying acute and late toxicity and focusing on mucositis CTC °III. Secondary endpoints consist of evaluation of efficacy (local control, disease-free survival) and toxicity (other than mucositis).

Initial results of the pilot project at the GSI/Darmstadt (treatment of skull base ACC) reported mucositis CTC °III or higher in only 6% of cases, with a recent update confirming these findings [18]. Assuming higher normal tissue sparing by increase of the heavy-ion part of the treatment, we would expect toxicity/mucositis rates unchanged or even lower.

## Methods/design

### Study design

The COSMIC trial is a prospective, non-randomized phase II feasibility trial evaluating acute mucositis ≥ CTCAE °III (v. 3.0) as the primary endpoint. Mucositis CTCAE °III is defined as confluent ulcerations or pseudomembranes within the oral cavity and bleeding with minor trauma leading to the inability of the patient to adequately aliment or hydrate orally.

### Study Characteristics

Combined IMRT and C12-heavy ion boost has previously been established as a treatment of choice in skull-base adenoid cystic carcinoma. However, based on the fact that local control seems to be dose-dependent, all types of salivary gland malignancies in the head and neck will probably benefit from high-LET radiation. Therefore, the combination of IMRT (50 Gy in 2 Gy/fraction) and C12-boost (24 GyE in 3 GyE/fraction) will be tested as to toxicity profile and efficacy.

### Study objectives

To evaluate feasibility and toxicity (with a focus on mucositis CTCAE °III (v. 3.0)) and efficacy of the treatment.

Incidence of mucositis ≥ CTCAE°III (v. 3.0) will be assessed as the primary endpoint of the trial, local control, disease-free survival, toxicity (incl. mucositis CTCAE °I-II and late toxicity at 3 years post RT)

### Sample size/number of subjects

Incidence of mucositis CTCAE °III in the initial data was 6% for adenoid cystic carcinoma mostly of the skull base. 29 patients with adneoid cystic carcinoma were treated with 54 Gy IMRT and 18 GyE carbon ions, two of these patients (i.e. 6.9%) showed mucositis CTCAE °III. Inclusion and exclusion criteria in COSMIC correspond to the initial patient selection; hence incidence of toxicity should also roughly correspond. Agreeing to an incidence of 6%, 49 patients have to be treated in order to observe at least one patient with mucositis CTCAE °III or higher with a probability of 95%. Assuming a drop-out rate of 10%, 54 patients need to be recruited.

### Patient selection

#### Inclusion criteria

• Histologically confirmed or surgically removed malignant tumour of the salivary glands (head and neck)

• Inoperable tumour

• G2/3

• Macroscopic or microscopic residual tumour (R2/R1) or

• ≥T3/T4 or

• perineural invasion (Pn+)

• written informed consent

• pts aged 18 - 80 years

• effective contraception for pts in childbearing age (< 12 months post beginning of menopause)

#### Exclusion criteria

• Prior radio- or chemotherapy for tumours of the head and neck

• Other previous malignancy within the past 5 years except prior, adequately treated basal cell carcinoma of the skin or pre-invasive carcinoma of the cervix

• Significant neurological or psychiatric condition including dementia or seizures or other serious medical condition prohibiting the patient's participation in the trial by judgement of the investigators

• Legal incapacity or limited legal capacity

• Positive serum/urine β-HCG/pregnancy

• Drug abuse

### Radiotherapy

#### Immobilisation/planning examinations

Patients are immobilized using individual thermoplastic head masks with thermoplastic shoulder fixation. Planning examinations consist of a planning CT scan (3 mm slice thickness) with the patient positioned in the individual fixation device and contrast-enhanced MRI for 3D image correlation.

#### Target volumes/dose prescription

CTV1 (carbon ion boost) includes the macroscopic tumour/prior tumour bed with special focus on the R1-area as well as respective neural pathways to the base of skull (cave: perineural invasion and skip lesions). For tumours of the parotid gland, the whole former parotid area is also included in the CTV1, if possible the mandibular joint is kept outside the CTV1. PTV1 consists of a 3 mm margin around the CTV1 but does not extend into critical organs at risk (i.e. brain stem, spinal cord).

We prescribe a dose of 24 GyE carbon ions in 3 GyE/fraction (5 fractions per week) to the CTV1, we aim at covering the CTV1 with the 95% prescription isodose. Treatment is given at the HIT (Heidelberg ion therapy centre) after inverse treatment planning in active beam application (raster-scanning method). Daily image guidance consists of orthogonal x-ray controls in treatment position.

CTV2 includes CTV1 with safety margins along typical pathways of spread. Only ipsilateral nodal levels (II and III) are includes, however, in case the primary tumour is/was located at midline or crossing midline, we cover bilateral nodal levels II and III. In case there is a pathological lymph node involvement, additional nodal level will be covered as indicated. CTV2 also encompasses the complete surgical operational area. The CTV2 also takes account for set-up variations, hence corresponds to the PTV2 (CTV2 = PTV2). Should the primary tumour be located within the parotid gland, also the parotid duct needs to be within the CTV2.

50 Gy IMRT (inversely planned step-and-shoot or tomotherapy technique) in 25 fractions (5 fractions per week) are prescribed to the CTV2 (coverage at least with the 90% prescription isodose) taking into account doses applied by daily image guidance with MV-cone-beam CT.

#### Supportive therapy

Patients do not routinely receive prophylactic feeding tubes, however if they uncommonly experience significant weight loss we will of course offer feeding tube insertion or parenteral feeding.

#### Treatment schedule/follow-up

**Figure 1:**
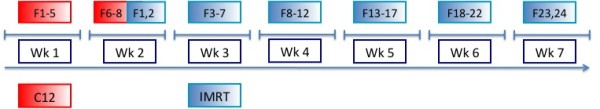
**trial flow-chart**.

After obtaining written informed consent, patients are included into the trial and receive their treatment planning investigations. Treatment usually starts with 8 fractions carbon ion (8 × 3 GyE C12) therapy followed by 27/28 fractions of IMRT corresponding to a total dose of approximately 74 GyE. Treatment duration is approximately 6-7 weeks (figure 1).

First follow-up examination including diagnostic, contrast-enhanced MRI will be carried out 6 weeks post completion of radiation treatment. Further controls including MRI are 3, 6, and 12 months thereafter, in 6 monthly intervals until 2 years post RT, then in yearly intervals. Abdominal ultrasound will be carried out q6 months, chest-CT q12 months. At each follow-up appointment, patients receive a symptom-oriented clinical examination, also patients' performance state (Karnofsky-Index), therapy-associated side effects as well as potential intercurrent therapy is recorded.

Patients are also encouraged to undergo regular check-ups incl. full ENT clinical examinations in regular intervals.

#### Proteomics and Genomics

For the proteomic examinations 30 mL venous blood will be collected from each subject prior to RT, at day 29, day 44 or 45, and at the 1^st ^and 2^nd ^follow-up visit. Thus, the overall volume of blood samples used for Proteomic/Genomic investigations will be approximately 200 mL. Following parameters/pathways will be investigated:

• In order to predict the efficacy of the therapy blood will be collected during therapy and follow-up to detect and correlate the levels of well known tumour- and angiogenesis markers (VEGF, TGF-Alpha, bFGF, IL8, k-ras, etc.) using Enzyme-Linked Immunosorbent Assay (ELISA).

• Further, platelet protein content (i.e. tumor angiogenesis growth factors and cytokines) will be analyzed using citrate blood samples and correlated with serum- and plasma- protein results.

In order to perform the genomic analysis, patients' blood samples are collected as indicated and RNA, miRNA and DNA isolation will be performed. Based on an established platform, linear RNA-amplification, labelling and hybridization on human genome wide oligo-arrays (transcriptome analysis) are planned. DNA samples are used to identify potential chromosomal aberrations or epigenetic alterations that might predict treatment response. RNA and miRNA samples are further analyzed by real time quantitative RT-PCR to confirm microarray data and to test a subset of clinical predictors.

The determinations of proteomic and genomic parameters will be carried out at the Department of Radiation Oncology in Heidelberg. No further genetic investigations on the blood collected during the study will be carried out.

#### Assessment of efficacy

Assessment of efficacy will be carried out by evaluation of imaging studies (MRI) at each follow-up. If applicable (in case of initial macroscopic tumour) tumour response will be evaluated according to the RECIST-criteria (19).

#### Trial organization/coordination

The COSMIC trial has been designed by the Department of Radiation Oncology, University of Heidelberg, and is carried out at the Heidelberg Ion Therapy Centre (HIT). It is an investigator-initiated trial; the Department of Radiation Oncology is responsible for co-ordination, overall trial management, registration (clinicaltrials.gov Identifier: NCT 01154270), database management, quality assurance, monitoring, and reporting.

#### Investigators

Patients are recruited by the Department of Radiation Oncology.

#### Adverse events

Adverse and serious adverse events are recorded using NCI common toxicity criteria for adverse events (CTCAEAE v. 3.0). Acute radiation effects are defined as effects occurring within 90 days from beginning of radiotherapy. Late effects are defined as effects observed thereafter. Safety analysis is performed with respect to frequency of serious adverse events and adverse events stratified by organ system, severity, causality.

#### Regular completion of the trial

Patient accrual is completed with inclusion of the last patient and should extend for approximately 2 years from trial initiation. Regular trial participation for each patient terminates 3 years post inclusion into the trial or the patient's death respectively.

#### Discontinuation of treatment

• Patient wish

• Medical condition necessitating treatment termination and withdrawal of the patient from the trial

• Pregnancy

• Lack of compliance

#### Premature termination of the trial

The trial can be prematurely closed or suspended by the LKP in following cases:

• Medical or ethical reasons relevantly affecting the risk-benefit relationship,

• Difficulties in recruitment of subjects suggest unjustifiable prolongation of the study timeline,

• Previously unexpected adverse events (in respect of their nature, severity, duration or outcome) occur with unjustifiable frequency,

• Expected adverse events occur with an unexpectedly high incidence,

• Relevant superiority of patients in one treatment arm of a comparable clinical trial,

• Legal authorities' decision

The Ethics Committee (EC) and the competent regulatory authorities will be informed about premature closure of the trial. Furthermore, the Ethics Committee(s) and competent regulatory authorities themselves may decide to stop or suspend the trial.

If the trial is closed prematurely, the trial material such as completed, partially completed, and blank CRFs will be returned to the coordinating investigator.

All involved investigators have to be informed immediately about a cessation or suspension of the trial. The decision is binding on all trial centers and investigators.

#### Ethics, informed consent, and safety

The final protocol was approved by the ethics committee of the University of Heidelberg Medical School (S-263/2009). The trial complies with the Helsinki Declaration in its recent German version, the Medical Association's professional code of conduct, principles of Good Clinical Practice (GCP) guidelines and the Federal Data Protection Act. It will be carried out in keeping with local legal and regulatory requirements. It is also subject to authorization by the German radiation protection authority (Bundesamt für Strahlenschutz: = BfS). Medical confidentiality and Federal Data Protection Act will be followed. Written informed consent is obtained from each patient in oral and written form.

## Discussion

Achieving local control in malignant salivary gland tumour remains challenging. Progress has been made by the introduction of novel radiotherapy techniques like fractionated stereotactic RT and IMRT, however, particle therapy (neutron and carbon ion therapy) result in the highest control rates in adenoid cystic carcinoma so far. Compared to neutron RT, carbon ion treatment comes at the cost of only mild side effects and is now permanently available at a new dedicated particle therapy facility (HIT). Considering the fact local control in salivary gland tumours is dose dependent, we believe treatment outcome can be improved for all malignant salivary gland histologies without increasing side-effects by a combination of IMRT (50 Gy) with a carbon ion boost (24 GyE) not only in incompletely resected or unresectable tumours but also in the R1-situation or perineural infiltration. The COSMIC trial was designed to address these issues in a prospective phase II trial. To our knowledge this is the first phase II trail combining particle therapy and IMRT.

## Competing interests

The authors declare that they have no competing interests.

## Authors' contributions

ADJ, MWM, CWK, and JD developed the study protocol and planned the trial. CWK is responsible for statistical considerations/basis of the trial. ADJ, AN, MWM are responsible for conducting and co-ordination of the trial as well as patient recruitment. All authors read and approved the final manuscript

## Pre-publication history

The pre-publication history for this paper can be accessed here:

http://www.biomedcentral.com/1471-2407/10/546/prepub
